# Molecular detection of pathogens in ticks and fleas collected from companion dogs and cats in East and Southeast Asia

**DOI:** 10.1186/s13071-020-04288-8

**Published:** 2020-08-15

**Authors:** Viet-Linh Nguyen, Vito Colella, Grazia Greco, Fang Fang, Wisnu Nurcahyo, Upik Kesumawati Hadi, Virginia Venturina, Kenneth Boon Yew Tong, Yi-Lun Tsai, Piyanan Taweethavonsawat, Saruda Tiwananthagorn, Sahatchai Tangtrongsup, Thong Quang Le, Khanh Linh Bui, Thom Do, Malaika Watanabe, Puteri Azaziah Megat Abd Rani, Filipe Dantas-Torres, Lenaig Halos, Frederic Beugnet, Domenico Otranto

**Affiliations:** 1 Dipartimento di Medicina Veterinaria, Università degli Studi di Bari, Bari, Italy; 2 Faculty of Veterinary and Agricultural Sciences, University of Melbourne, Melbourne, Australia; 3 School of Animal Science and Technology, Guangxi University, Nanning, China; 4 Faculty of Veterinary Medicine, Gadjah Mada University, Yogyakata, Indonesia; 5 Faculty of Veterinary Medicine, IPB University, Bogor, Indonesia; 6 College of Veterinary Science & Medicine, Central Luzon State University, Nueva Ecija, Philippines; 7 Animal & Avian Veterinary Clinic, Singapore, Singapore; 8 Department of Veterinary Medicine, National Pingtung University of Science and Technology, Pingtung, Taiwan; 9 Faculty of Veterinary Science, Chualalongkorn University, Bangkok, Thailand; 10 Faculty of Veterinary Medicine, Chiang Mai University, Chiang Mai, Thailand; 11 Faculty of Animal Science & Veterinary Medicine, Nong Lam University, Ho Chi Minh city, Vietnam; 12 Faculty of Veterinary Medicine, Vietnam National University of Agriculture, Hanoi, Vietnam; 13 Biodiversity Conservation and Tropical Disease Research Institute, Hanoi, Vietnam; 14 Faculty of Veterinary Medicine, Universiti Putra Malaysia, Serdang, Malaysia; 15 Department of Immunology, Aggeu Magalhães Institute, Recife, Brazil; 16 Boehringer Ingelheim Animal Health, Lyon, France; 17 Faculty of Veterinary Sciences, Bu-Ali Sina University, Hamedan, Iran

**Keywords:** Ticks, Fleas, Dogs, Cats, Companion animals, Asia, Vector-borne pathogens, Zoonotic

## Abstract

**Background:**

Ticks and fleas are considered amongst the most important arthropod vectors of medical and veterinary concern due to their ability to transmit pathogens to a range of animal species including dogs, cats and humans. By sharing a common environment with humans, companion animal-associated parasitic arthropods may potentially transmit zoonotic vector-borne pathogens (VBPs). This study aimed to molecularly detect pathogens from ticks and fleas from companion dogs and cats in East and Southeast Asia.

**Methods:**

A total of 392 ticks and 248 fleas were collected from 401 infested animals (i.e. 271 dogs and 130 cats) from China, Taiwan, Indonesia, Malaysia, Singapore, Thailand, the Philippines and Vietnam, and molecularly screened for the presence of pathogens. Ticks were tested for *Rickettsia* spp., *Anaplasma* spp., *Ehrlichia* spp., *Babesia* spp. and *Hepatozoon* spp. while fleas were screened for the presence of *Rickettsia* spp. and *Bartonella* spp.

**Result:**

Of the 392 ticks tested, 37 (9.4%) scored positive for at least one pathogen with *Hepatozoon canis* being the most prevalent (5.4%), followed by *Ehrlichia canis* (1.8%), *Babesia vogeli* (1%), *Anaplasma platys* (0.8%) and *Rickettsia* spp. (1%) [including *Rickettsia* sp. (0.5%), *Rickettsia asembonensis* (0.3%) and *Rickettsia felis* (0.3%)]. Out of 248 fleas tested, 106 (42.7%) were harboring at least one pathogen with *R. felis* being the most common (19.4%), followed by *Bartonella* spp. (16.5%), *Rickettsia asembonensis* (10.9%) and “*Candidatus* Rickettsia senegalensis” (0.4%). Furthermore, 35 *Rhipicephalus sanguineus* ticks were subjected to phylogenetic analysis, of which 34 ticks belonged to the tropical and only one belonged to the temperate lineage (*Rh. sanguineus* (*sensu stricto*)).

**Conclusion:**

Our data reveals the circulation of different VBPs in ticks and fleas of dogs and cats from Asia, including zoonotic agents, which may represent a potential risk to animal and human health.
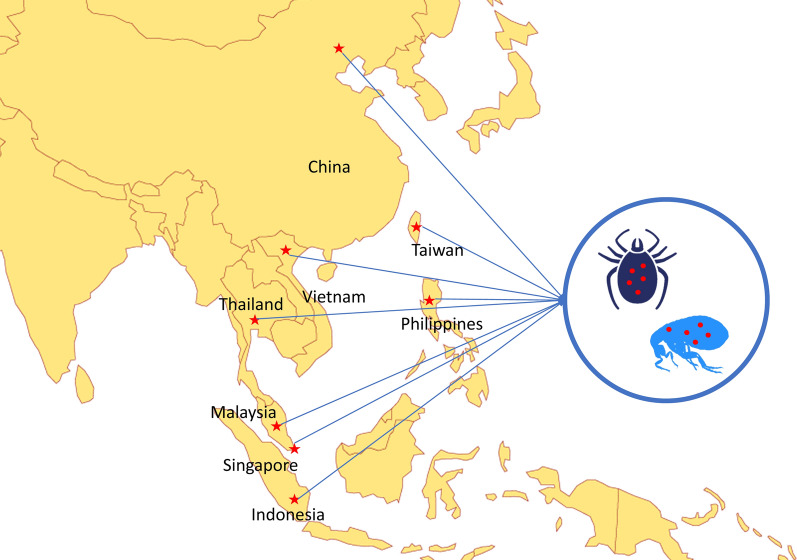

## Background

Vector-borne diseases are caused by bacteria, viruses, protozoa and helminths transmitted by arthropod vectors, including ticks and fleas, worldwide [1]. For instance, *Rhipicephalus sanguineus* (*sensu lato*) ticks play an important role in the transmission of many pathogens to dogs (e.g. *Ehrlichia canis*, *Rickettsia conorii*, *Rickettsii rickettsia*, *Babesia vogeli* and *Hepatozoon canis*), some of which may also infect humans [2–4]. The cat flea *Ctenocephalides felis* is the primary vector of *Bartonella henselae*, the main causative agent of cat-scratch disease [5,6], and is also considered as vector of *Rickettsia felis* [7].

East (EA) and Southeast Asia (SEA) are among the world’s fastest-growing economic regions [8], which also resulted in a rise in the number of companion dogs and cats [9]. Companion dogs and cats live in close association with humans, potentially carrying ticks and fleas into human settlements. A large-scale survey conducted in EA and SEA reported that 22.3% of dogs and 3.7% of cats were infested by ticks, while 14.8% of dogs and 19.6% of cats were infested by fleas [10]. The most common flea species parasitizing dogs and cats in EA and SEA is *C. felis*, with *Ctenocephalides orientis* being increasingly observed in dogs [10, 11]. *Rhipicephalus sanguineus* (*s.l.*), *Rhipicephalus haemaphysaloides* and *Haemaphysalis longicornis* represent the most common tick species reported in dogs and cats [10–14]. These tick species are responsible for the transmission of several species of apicomplexan protozoa of the genus *Babesia. Babesia vogeli* was reported in cats in Thailand and China [15, 16] and widely reported in dogs in EA and SEA, including China, Cambodia, Thailand, the Philippines and Malaysia [17–20]. Additionally, dogs from Taiwan, Malaysia, China, and Singapore [10, 21, 22] were also diagnosed with *Babesia gibsoni* infection. Other apicomplexan parasite commonly found in dogs across this region is *H. canis*, which is transmitted by ingestion of *Rh. sanguineus* (*s.l.*). This protozoan is commonly found in dogs from Thailand, Taiwan, China, Cambodia, Malaysia, Vietnam and the Philippines [10, 17, 18, 23–26] and in cats from Thailand and the Philippines [10, 24]. Of the tick-borne anaplasmataceae bacteria, *Anaplasma platys* was found in dogs from Malaysia [22] and cats from Thailand [27]. Apart from tick-borne pathogens, flea-borne pathogens are also increasingly recognized as important pathogenic agents to animals and humans. For instance, *R. felis*, the etiological agent of flea-borne spotted fever in humans, has been detected in dogs from Cambodia and China [18, 28] and in *C. felis* from Taiwan, Laos, and Malaysia [29, 30]. Other zoonotic flea-borne pathogens such as *B. henselae* and *Bartonella clarridgeiae*, agents of cat-scratch disease, were molecularly detected in cats and their fleas from the Philippines, Indonesia, Singapore, Thailand, Malaysia and China with the prevalence ranging from 10 to 60% [31–36].

Despite previous scientific investigations reported the circulation of vector-borne pathogens (VBPs) in dogs and cats in EA and SEA, there is a lack of similar studies conducted in their associated ticks and fleas. Therefore, the present study aimed to provide an overview of the pathogens circulating in ticks and fleas from companion dogs and cats in EA and SEA.

## Methods

### Samples collection and DNA isolation

Of the 2381 privately-owned animals examined (i.e. 1229 dogs and 1152 cats), ticks and fleas were collected from 401 infested animals (i.e. 271 dogs and 130 cats) from China, Taiwan, Indonesia, Malaysia, Singapore, Thailand, the Philippines and Vietnam under the context of a previous multicenter survey [10]. Ticks and fleas were collected and placed in labelled tubes individualized per host, containing 70% ethanol. Ticks and fleas (20%) were randomly selected from each tick/flea species and from each infested animal in all studied countries, giving a total number of 392 ticks (i.e. 377 *Rh. sanguineus* (*s.l.*), 3 *Rh. haemaphysaloides*, 7 *H. longicornis*, 2 *Haemaphysalis wellingtoni*, 1 *Haemaphysalis hystricis*, 1 *Haemaphysalis campanulata* and 1 *Ixodes* sp.) from 248 animals (39 cats and 209 dogs) and 248 fleas (i.e. 209 *C. felis*, 38 *C. orientis* and 1 *Xenopsylla cheopis*) from 213 animals (104 cats and 109 dogs) were subjected to DNA isolation individually. Data on the molecular identification of these ticks and fleas are available elsewhere (see Table 4 in Colella et al. [10]) Genomic DNA was isolated according to the procedures previously described [10, 37].

### Molecular detection and phylogenetic analysis of pathogens

Tick DNA samples were tested for the presence of apicomplexan protozoa (i.e. *Babesia* spp., *Hepatozoon* spp.), *Anaplasmataceae* (i.e. *Anaplasma* spp., *Ehrlichia* spp.) and *Coxiella burnetii* by conventional PCR (cPCR). Flea DNA samples were tested by using real-time PCR for *Bartonella* spp. The presence of *Rickettsia* spp. was also screened in both tick and flea samples. In particular, the first cPCR amplified a portion of citrate synthase (*gltA*) gene, which is presented in all members of the genus *Rickettsia*. Positive samples were then subjected to a second cPCR, which amplified a fragment of the outer membrane protein (*ompA*) of the spotted fever group (SFG) rickettsiae. All primers and PCR protocols used for the detection of VBPs are summarized in Table 1. For all reactions, DNA of pathogen-positive samples served as a positive control. Amplified cPCR products were examined on 2% agarose gels stained with GelRed (VWR International PBI, Milan, Italy) and visualized on a GelLogic 100 gel documentation system (Kodak, New York, USA). The cPCR amplicons were sequenced using the Big Dye Terminator v.3.1 chemistry in a 3130 Genetic analyzer (Applied Biosystems, California, USA). Nucleotide sequences were edited, aligned and analyzed using the BioEdit 7.0 software and compared with those available in the GenBank database using Basic Local Alignment Search Tool (http://blast.ncbi.nlm.nih.gov/Blast.cgi).

**Table 1:** Primers, target genes and PCR conditions used in this study

Pathogen	Primer (5’-3’)	Target gene	Product size (bp)	PCR protocol	Reference
*Babesia* spp./*Hepatozoon* spp.	Piroplasmid-F: CCAGCAGCCGCGGTAATTC	*18S* rRNA	350–400	95 °C for 10 min initial denaturation, followed by 35 cycles of 95 °C for 30 s, 64 °C for 20 s, 72 °C for 20 s, then 72 °C for 7 min for the final elongation	[38]
	Piroplasmid-R: CTTTCGCAGTAGTTYGTCTTTAACAAATCT				
*Ehrlichia* spp*./ Anaplasma* spp.	EHR16SD: GGTACCYACAGAAGAAGTCC	*16S* rRNA	345	95 °C for 10 min initial denaturation, followed by 35 cycles of 95 °C for 30 s, 60 °C for 30 s, 72 °C for 30 s, then 72 °C for 10 min for the final elongation	[39]
	EHR16SR: TAGCACTCATCGTTTACA GC				
*Coxiella burnetii*	Trans-1: TATGTATCCACCGTAGCCAGT	*IS1111a*	687	95 °C for 10 min initial denaturation, followed by 35 cycles of 95 °C for 30 s, 64 °C for 60 s, 72 °C for 60 s, then 72 °C for 7 min for the final elongation	[40]
	Trans-2: CCCAACAACACCTCCTTATTC				
*Bartonella* spp.	ssrA-F: GCTATGGTAATAAATGGACAATGAAATAA	*ssrA*	301	95 °C for 2 min initial denaturation, followed by 45 cycles of 95 °C for 15 s, 60 °C for 60 s	[41]
	ssrA-R: GCTTCTGTTGCCAGGTG				
	Probe: FAM-ACCCCGCTTAAACCTGCGACG				
*Rickettsia* spp.	CS-78F: GCAAGTATCGGTGAGGATGTAAT	*gltA*	401	95 °C for 10 min initial denaturation, followed by 40 cycles of 95 °C for 30 s, 58 °C for 30 s, 72 °C for 40 s, then 72 °C for 7 min for the final elongation	[42]
	CS-323R: GCTTCCTTAAAATTCAATAAATCAGGAT				
Spotted fever group rickettsiae	Rr190.70F: ATGGCGAATATTTCTCCAAAA	*ompA*	632	95 °C for 10 min initial denaturation, followed by 35 cycles of 94 °C for 40 s, 58 °C for 30 s, 72 °C for 45 s, then 72 °C for 10 min for the final elongation	[43]
	Rr190.701R: GTTCCGTTAATGGCAGCATCT				

To assess the genetic variation of *Rh. sanguineus* (*s.l.*) and *Rickettsia* spp., the mitochondrial *16S* rDNA sequences of *Rh. sanguineus* (*s.l.*) ticks generated previously [10] as well as the *gltA* and *ompA* gene sequences of *Rickettsia* spp. generated herein were subjected to phylogenetic analysis. Phylogenetic relationship was inferred by Maximum Likelihood (ML) method after selecting the best-fitting substitution model. Evolutionary analysis was conducted on 8000 bootstrap replications using the MEGA 7 software [44].

### Statistical analysis

The percentage of detected pathogens was calculated and 95% confidence intervals (95% CI) (by the modified Wald method) were estimated by using Quantitative Parasitology 3.0 software [45]. Fisher’s exact test was performed to analyze statistically significant differences in the detection of pathogens in fleas and ticks, and in the distribution of different *Rickettsia* spp. among different flea species using SPSS 16.0 software. Differences were considered significant at *P *< 0.05.

## Results

The occurrence of VBPs has been detected in ticks and fleas, with a higher number of fleas in which at least one pathogen was detected compared to ticks (Fisher’s exact test, *P* < 0.001). Of the 392 ticks tested, 37 (9.4%; 95% CI: 6.9‒12.8%) scored positive for at least one pathogen with *H. canis* being the most prevalent (5.4%; 95% CI: 3.5‒8.1%), followed by *E. canis* (1.8%; 95% CI: 0.8‒3.7%), *B. vogeli* (1%; 95% CI: 0.3‒2.7%), *Rickettsia* spp. (1%; 95% CI: 0.3‒2.7%) and *A. platys* (0.8%; 95% CI: 0.2‒2.3%). Co-infection of *A. platys* and *B. vogeli* was detected in one *Rh. sanguineus* (*s.l.*), whereas none of the ticks tested positive for *C. burnetii* (Table 2). Out of 248 fleas tested, 106 (42.7%; 95% CI: 36.7‒49.0%) were harboring at least one pathogen with *R. felis* being the most common (19.4%; 95% CI: 14.9‒24.8%), followed by *Bartonella* spp. (16.5%; 95% CI: 12.4‒21.7%), *Rickettsia asembonensis* (10.9%; 95% CI: 7.6‒15.4%) and “*Candidatus* Rickettsia senegalensis” (0.4%; 95% CI: < 0.0001‒2.5%) (Table 3). *Rickettsia felis* was mostly detected in *C. felis*, whereas *C. orientis* mainly harbored *R. asembonensis* (*P* < 0.001).

**Table 2:** Pathogens detected in ticks according to their species, developmental stage, sex and host in East and Southeast Asia

	*Anaplasmataceae*		*Rickettsia* spp.			Apicomplexan protozoans	
	*Anaplasma platys*	*Ehrlichia canis*	*Rickettsia felis*	*Rickettsia asembonensis*	*Rickettsia* sp.	*Babesia vogeli*	*Hepatozoon canis*
China (*n* = 28)							1
C; *Haemaphysalis longicornis* (1L)							
D; *Haemaphysalis campanulata* (1L)							
D; *Haemaphysalis longicornis* (3L, 1N, 1F, 1M)							
D; *Rhipicephalus sanguineus* (*s.l.*) (3L, 7N, 5F, 5M)							1F
Indonesia (*n* = 79)		2		1			
C; *Rhipicephalus sanguineus* (*s.l.*) (2N, 6F, 2M)		1F					
D; *Rhipicephalus sanguineus* (*s.l.*) (2L, 10N, 26F, 30M)		1F		1F			
D; *Haemaphysalis wellingtoni* (1N)							
Malaysia (*n* = 3)	2					1	
D; *Rhipicephalus sanguineus* (*s.l.*) (1F, 2M)	1F, 1M					1M	
The Philippines (*n* = 90)		2	1				12
C; *Rhipicephalus sanguineus* (*s.l.*) (6N, 8F, 13M)		1N, 1F					1N, 1M
D; *Rhipicephalus sanguineus* (*s.l.*) (1L, 6N, 23F, 33M)			1M				1N, 2F, 7M
Singapore (*n* = 4)							
C; *Rhipicephalus sanguineus* (*s.l.*) (1N, 1F, 2M)							
Taiwan (*n* = 25)	1				2		1
C; *Ixodes* sp. (1F)^a^							
D; *Rhipicephalus haemaphysaloides* (1F, 2M)					1F, 1M		
D; *Rhipicephalus sanguineus* (*s.l.*) (2N, 9F, 10M)	1M						1F
Thailand (*n* = 46)		3					1
D; *Haemaphysalis hystricis* (1F)							
D; *Haemaphysalis wellingtoni* (1L)							
D; *Rhipicephalus sanguineus* (*s.l.*) (2L, 3N, 20F, 9M)		1F, 2M					1F
Vietnam (*n* = 117)						3	6
D; *Rhipicephalus sanguineus* (*s.l.*) (18N, 48F, 51M)						2F, 1M	5F, 1M
Total	3	7	1	1	2	4	21

*Abbreviations*: C, cat; D, dog; L, larva; N, nymph; M, adult male; F, adult female

^a^This female tick was reported as “*Ixodes* sp.” in [10]. Following reassessment of photomicrography images of this tick by one of the co-authors (F.D.-T.) the following morphological features were observed: auriculae and cornua present; porose area small, not contiguous, hypostome with a 2/2 dental formula on almost the entire hypostome; coxa I with slight internal spur, coxae III and IV each with external spur; syncoxae present on coxae I and II; trochanters lacking spurs. As such, this female shares several morphological features with *Ixodes ovatus* [46], but genetic data from a partial *16S* rDNA sequence (percent identity: 90.7% with U95900) suggest that this may belong to a distinct species

**Table 3:** Pathogens detected in fleas according to their species, developmental stage, sex and host in East and Southeast Asia

	*Rickettsia* spp.			*Bartonella* spp.
	*R. felis*	*R. asembonensis*	“*Ca.* R. senegalensis”	
China (*n* = 17)	1			2
C; *Ctenocephalides felis* (8F, 2M)	1F			2F
D; *Ctenocephalides felis* (7F)				
Indonesia (*n* = 48)	12	9		21
C; *Ctenocephalides felis* (4F, 24M)	7F, 2M			18F
C; *Xenopsylla cheopis* (1M)				1M
D; *Ctenocephalides felis* (6F, 1M)	2F			2F
D; *Ctenocephalides orientis* (9F, 3M)	1M	7F, 2M		
Malaysia (*n* = 7)				
C; *Ctenocephalides felis* (4F, 1M)				
D; *Ctenocephalides felis* (2F)				
The Philippines (*n* = 90)	20	9		4
C; *Ctenocephalides felis* (24F, 9M)	4F, 3M			3F, 1M
D; *Ctenocephalides felis* (34F, 13M)	12F, 1M	1F		
D; *Ctenocephalides orientis* (9F, 1M)		8F		
Singapore (*n* = 2)				2
C; *Ctenocephalides felis* (1M)				1M
C; *Ctenocephalides orientis* (1F)				1F
Taiwan (*n* = 24)	8			4
C; *Ctenocephalides felis* (10F, 4M)	3F, 2M			3F, 1M
D; *Ctenocephalides felis* (6F, 3M)	3F			
D; *Ctenocephalides orientis* (1F)				
Thailand (*n* = 21)	1	4	1	5
C; *Ctenocephalides felis* (4F, 1M)				2F
D; *Ctenocephalides felis* (7F, 4M)	1F			1F
D; *Ctenocephalides orientis* (2F, 3M)		2F, 2M	1M	1F, 1M
Vietnam (*n* = 39)	6	5		3
C; *Ctenocephalides felis* (14F, 5M)	3F, 1M			3F
D; *Ctenocephalides felis* (8F, 3M)	2F			
D; *Ctenocephalides orientis* (7F, 2M)		4F, 1M		
Total	48	27	1	41

*Abbreviations*: C, cat; D, dog; F, female; M, male

Representative nucleotide sequences for each detected pathogen displayed 99.4‒100% identity with those available in GenBank database. In particular, *A. platys* nucleotide sequences (*n* = 3) revealed 99.6‒100% identity with KU500914 (host: *Canis lupus familiaris*; origin: Malaysia), *H. canis* (*n* = 20) 99.7‒100% identity with DQ519358 (host: C. *lupus familiaris*; origin: Thailand), *E. canis* (*n* = 7) and *B. vogeli* (*n* = 4) 100% identical to MN227484 (host: *C. lupus familiaris*; origin: Iraq) and KX082917 (host: *C. lupus familiaris*; origin: Angola), respectively.

For *Rickettsia* spp. detection in ticks, the *gltA* sequence identified in one tick showed 99.7% identity with *R. asembonensis* (GenBank: KY445723; host: *C. felis*; origin: Brazil) and another was identical to *R. felis* (100% nucleotide identity with MG845522 (host: *C. felis*; origin: Chile) and 99.4% nucleotide identity with *R. felis* strain URRWXCal2; GenBank: CP000053 (source: cultivation; origin: USA)). The *ompA* gene amplification was successful for two samples which showed 100% nucleotide identity with unidentified *Rickettsia* sp. (GenBank: EF219467; host: *R. haemaphysaloides*; origin: Taiwan), which was related (98.3%) to *Rickettsia rhipicephali* (GenBank: U43803; host: *C. felis*; origin: USA).

For *Rickettsia* spp. detection in fleas, amplification of a portion of the *gltA* gene was positive from 76 fleas. The partial sequence of the *ompA* gene was successfully obtained from 17 of the 76 *gltA*-positive *C. felis* fleas. All of those *ompA* gene sequences were 100% identical to *R. felis* strain URRWXCal2 (GenBank: CP000053; source: cultivation; origin: USA). Sequence analysis of the *gltA* genes fragment from the other 59 *Rickettsia* positive *C. felis* fleas revealed that the 31 sequences obtained had 99.4% nucleotide identity with *R. felis* strain URRWXCal2 (GenBank: CP000053; source: cultivation; origin: USA), 27 sequences 99.7% with *R. asembonensis* (GenBank: KY445723; host: *C. felis*; origin: Brazil) and one 100% with “*Ca.* R. senegalensis” (GenBank: MK548197; host: *C. felis*; origin: Colombia).

**Fig. 1:**
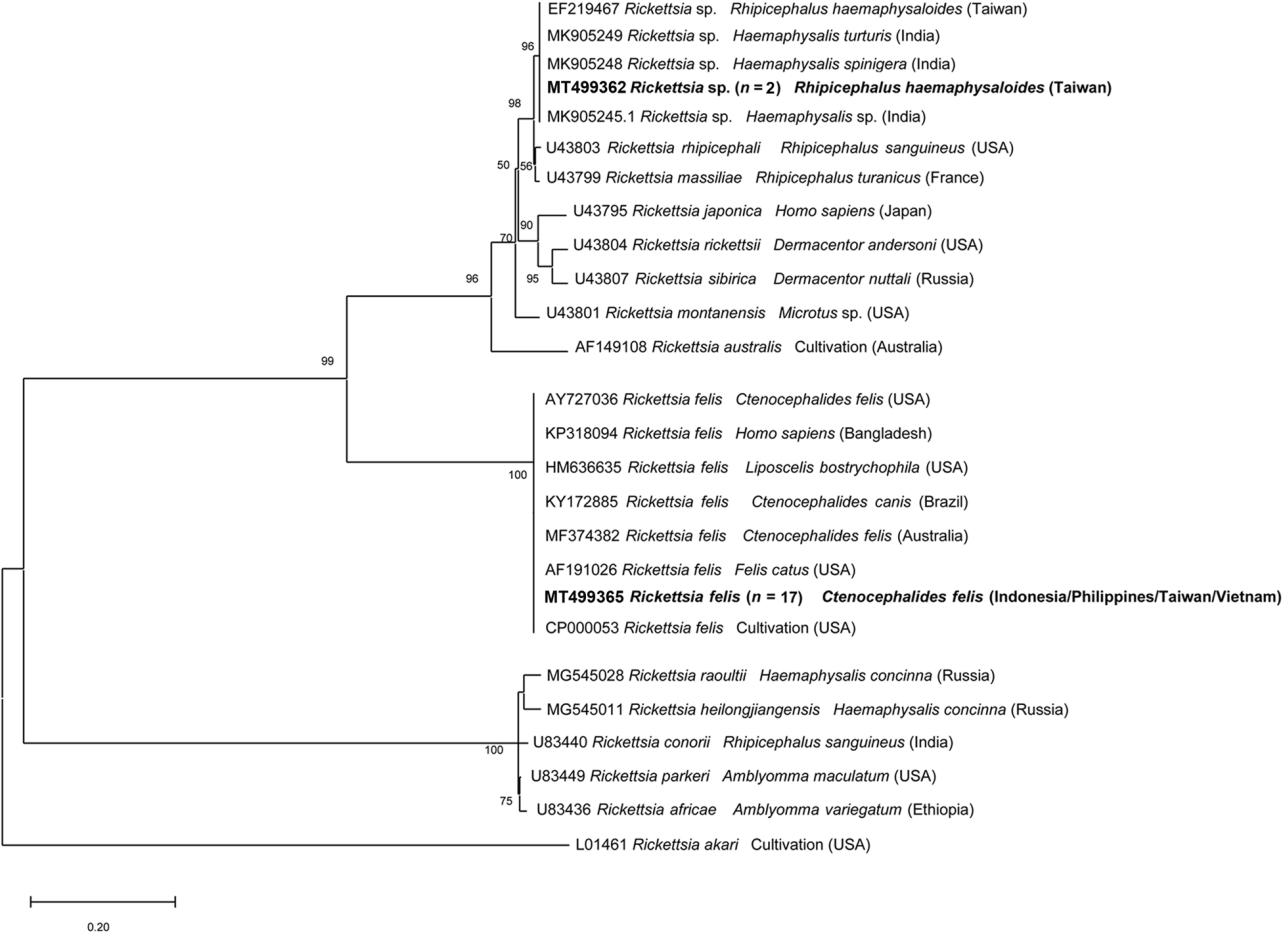
Phylogenetic relationships of *Rickettsia* spp. isolated in this study (in bold) to other *Rickettsia* spp. based on partial sequences of the *ompA* gene. The analyses were performed using a Maximum Likelihood method with Tamura 3-parameter model. A discrete Gamma distribution was used to model evolutionary rate differences among sites. GenBank accession number, isolation source and country of origin are presented for each sequence

**Fig. 2:**
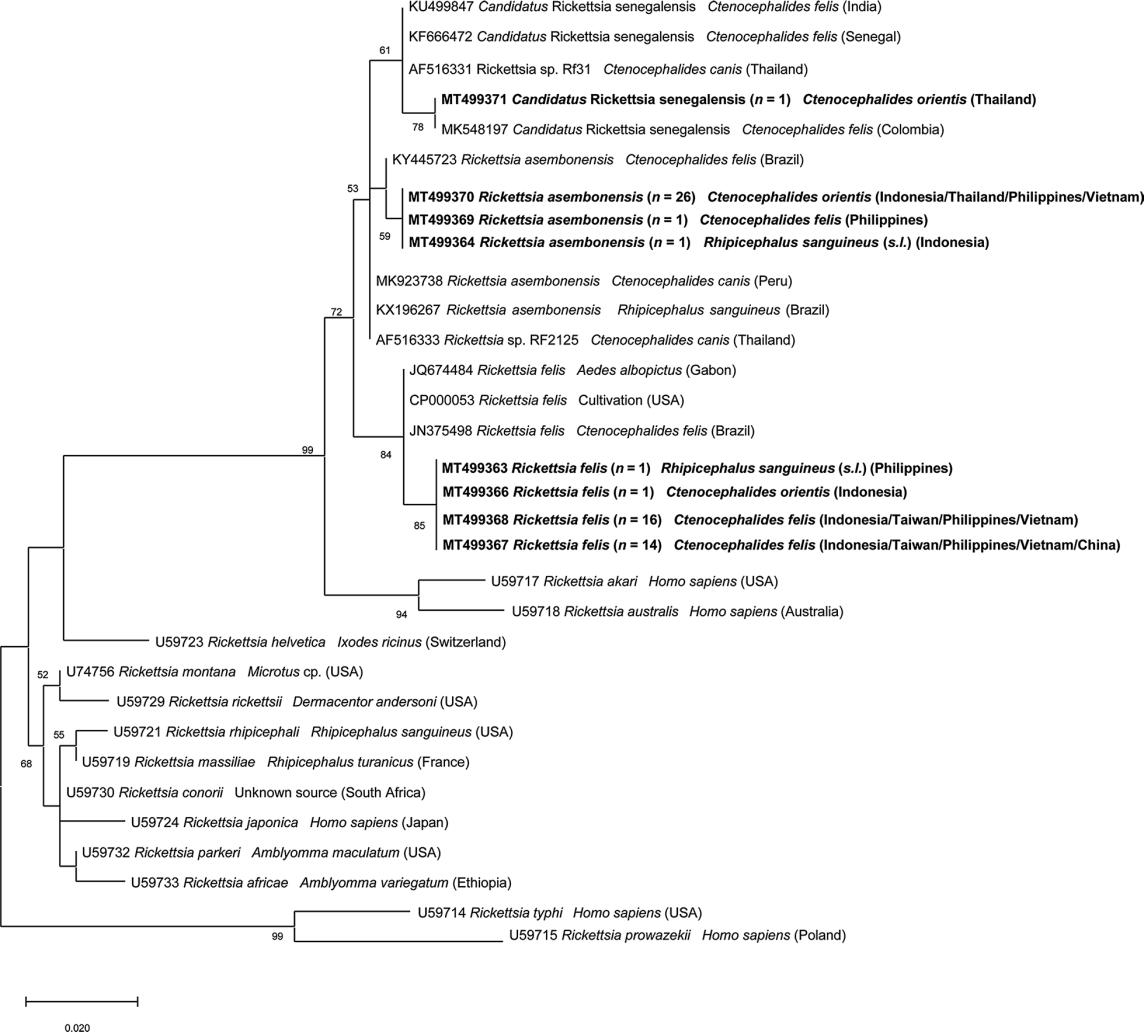
Phylogenetic relationships of *Rickettsia* spp. isolated in this study (in bold) to other *Rickettsia* spp. based on partial sequences of the *gltA* gene. The analyses were performed using a Maximum Likelihood method with Tamura 3-parameter model. A discrete Gamma distribution was used to model evolutionary rate differences among sites. GenBank accession number, isolation source and country of origin are presented for each sequence

The phylogenetic tree based on the partial *ompA* gene sequences showed that all *R. felis* isolated from fleas were assembled together in one cluster, whereas *Rickettsia* sp. isolated from ticks clustered with *R. rhipicephali* and *Rickettsia massiliae* (Fig 1). In the *gltA* tree, phylogenetical analysis revealed that *R. felis*, *R. asembonensis* and “*Ca.* R. senegalensis” herein detected were formed together in a well-supported sister cluster include other *R. felis*-like organisms (RFLOs), close to the cluster of *Rickettsia australis* and *Rickettsia akari* (Fig. 2).

**Fig. 3:**
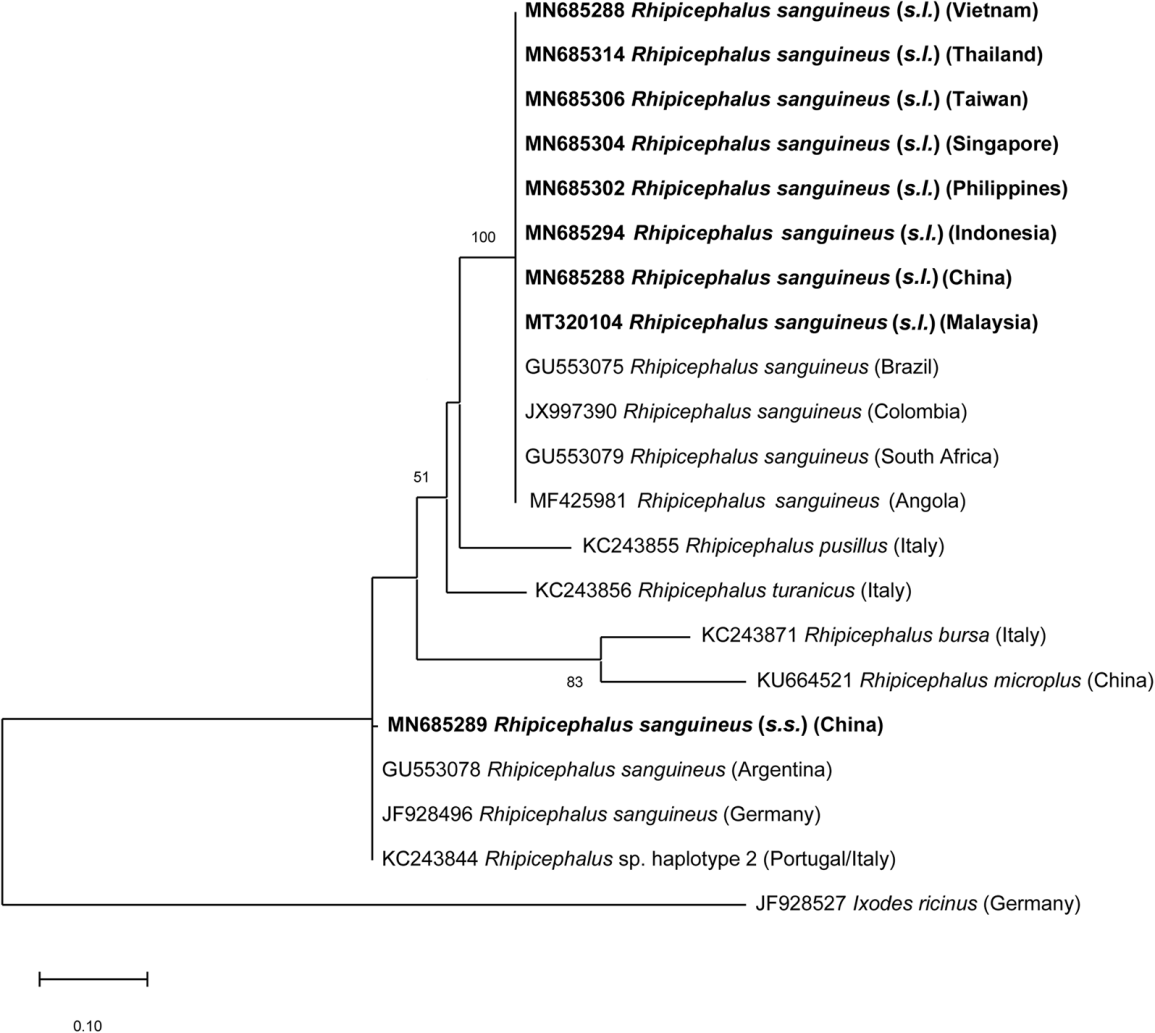
Phylogenetic relationships of the present *Rhipicephalus sanguineus* (*s.l*.) sequences (in bold) to other *Rhipicephalus* spp. based on a portion of the mitochondrial *16S* rRNA gene. As most sequences (*n* = 34) were identified as belonging to the tropical lineage *Rh. sanguineus* (*s.l*.), representatives were selected for each country. The analyses were performed using a Maximum Likelihood method with Tamura 3-parameter model. A discrete Gamma distribution was used to model evolutionary rate differences among sites. GenBank accession number, isolation source and country of origin are presented for each sequence

The ML tree of 35 representative mitochondrial *16S* rDNA sequences of *Rh. sanguineus* (*s.l.*) gene showed that 34 sequences were identical to each other and identified as belong to the tropical lineage of *Rh. sanguineus* (*s.l.*) (100% identity with GU553075; origin: Brazil). One sequence from a tick collected from a dog in Beijing (northeast China) clustered with *Rh. sanguineus* (sensu stricto) (100% identity with GU553078; origin: Argentina) (Fig. 3).

Representative sequences of pathogens detected in this study were deposited in the GenBank database under the accession numbers MT499354-MT499356 (*H. canis*), MT499357 (*B. vogeli*), MT499358 and MT499359 (*A. platys*), MT499360 and MT499361 (*E. canis*), MT499362 (*Rickettsia* sp.), MT499363-MT499367 (*R. felis*), MT499368-MT499370 (*R. asembonensis*) and MT499371 (“*Ca.* R. senegalensis”).

## Discussion

The results of this study reveal the presence of several pathogens in ticks (e.g. *A. platys*, *B. vogeli*, *E. canis*, *H. canis* and *Rickettsia* spp.) and fleas (e.g. *Rickettsia* spp. and *Bartonella* spp.) collected from dogs and cats in EA and SEA. The relatively low occurrence of pathogens herein detected in ticks is consistent with previous surveys conducted in ticks infesting owned dogs in Asia [13, 26, 47–49]. Conversely, the occurrence of VBPs is higher in ticks collected from stray animals [24]. Although *B. gibsoni* was identified in dogs from China (2.3%; [10]), none of the tested ticks from these dogs was found positive for this parasite. The absence of *B. gibsoni* in tick populations is probably due to the low number of *H. longicornis* and *H. hystricis*, which are recognized as vectors of this pathogen [50, 51]. The infection of *E. canis* (14.8% by serology) and *H. canis* (1.6% by cPCR) in host populations [10], along with the detection of these pathogens in *Rh. sanguineus* (*s.l.*) in the sampling areas support the vector role of this tick species in the transmission of these VBPs in this region [11, 14]. The finding of *A. platys* in dogs (7.1% by serology; [10]) and in *Rh. sanguineus* (*s.l.*) further suggests its vector competence for this pathogen. Additionally, the detection of *R. felis* and *R. asembonensis* in *Rh. sanguineus* (*s.l.*) is similar to previous results in Chile [52], Brazil [53] and Malaysia [54], consequently giving more concern about the role of *Rh. sanguineus* (*s.l.*) in the transmission of *Rickettsia* spp. other than *R. conorii*, *R. massiliae* and *R. rickettsii* [4, 55]. *Rickettsia* sp. sequences herein obtained from *Rh. haemaphysaloides* are identical to one previously generated from the same tick species in Taiwan (named *Rickettsia* sp. TwKM01) [56]. This genotype and its closest related species *R. rhipicephali* remain of unknown pathogenicity to mammals [56, 57]. Additionally, the vector role of *Rh. haemaphysaloides* needs further investigations since it was found harboring multiple pathogens such as *R. rhipicephali*, *A. platys*, *E. canis*, *B. gibsoni* [13, 58].

Of the detected VBPs, *R. felis* stood out as the most important due to its wide distribution, association with various arthropods, and importance as an emerging zoonotic pathogen [59]. In Asia, the first human case of flea-borne spotted fever attributed to *R. felis* was detected in the Thai-Myanmar border [60], since then several cases of human infection have been documented in Taiwan [61], Thailand [62], Laos [63], Vietnam [64] and Indonesia [65]. Although *R. felis* was detected in many arthropods, including non-hematophagous insect (i.e. the book louse *Liposcelis bostrychophila*) [66], the distribution of this rickettsia is highly affiliated with the distribution of *C. felis* [59]. *Ctenocephalides felis* is the most well-recognized vector of this rickettsia, which is transmitted transovarially and transstadially in the fleas [67], with dogs as proven mammalian reservoir hosts [68]. The high occurrence of *R. felis* in *C. felis* along with the high relative frequency of this flea species in host populations (65.1% in dogs and 98.7% in cats) [10] emphasizes the risk of *R. felis* infection in animals and humans.

The detection of *R. asembonensis* only from fleas collected on dogs (mainly *C. orientis* but in one case in *C. felis*) may suggest that dogs could act as amplifying hosts of this rickettsia, as they do for *R. felis* [68]. *Rickettsia asembonensis* is the most well-characterized genotype of RFLOs [69]. This rickettsia was initially described in fleas from dogs and cats in Kenya [70] and was then reported in various arthropods worldwide [69]. In Asia, *R. asembonensis* was also found in *C. orientis* from dogs [54] and in macaques from Malaysia [71]. Additionally, *Rickettsia* sp. RF2125, a genotype highly related to *R. asembonensis*, was reported with high incidence in *C. orientis* from India and Thailand [72, 73], and was also found in a febrile patient from Malaysia [74]. Moreover, *R. felis*, *R. asembonensis* and “*Ca.* R. senegalensis” clustered in the SFG rickettsiae clade (Fig. 3), and while *R. felis* is a recognized pathogen [67], the pathogenicity of other RFLOs is currently unknown.

Besides acting as vectors of *Rickettsia* spp., fleas have been well-recognized as vectors of *Bartonella* spp. [6, 75]. The occurrence of *Bartonella*-positive fleas in our study was slightly lower than previous investigations in Laos [30], Malaysia [35] and Thailand [76]. Nevertheless, the occurrence of the two common *Bartonella* spp. (i.e. *B. henselae* and *B. clarridgeiae*) in dogs and cats from EA and SEA is relatively high; up to 60% [18, 31, 76, 77]. Additionally, *B. henselae* infection in humans is usually associated to previous exposure to cats or cat fleas [78], emphasizing the role of cat fleas in *Bartonella* spp. transmission between animals and humans.

In the present study, all tested tick specimens were negative for *C. burnetii* although this pathogen was detected in *Rh. sanguineus* (*s.l.*) from dogs in Malaysia [79], in dogs in Taiwan [80] and in humans from Thailand [81]. Additionally, *Coxiella*-like endosymbionts were strongly associated with *Rh. sanguineus* (*s.l.*) tropical lineage [82], although the role of these endosymbionts in the biology and vectoral capacity of this tick lineage needs further investigation.

Finally, the genetic lineage of *Rh. sanguineus* (*s.l.*) from EA and SEA was investigated based on the *16S* rDNA sequences. The finding of *Rh. sanguineus* (*s.s.*) in Beijing, a cold area, along with the existence of the tropical lineage in warmer localities, agreed with previous studies, which indicated that the tropical lineage is present in areas with annual mean temperature > 20°C, whereas the temperate lineage occurs in areas with annual mean temperature < 20°C [83]. This information is also relevant from a pathogen transmission perspective, considering that different *Rh. sanguineus* (*s.l.*) lineages may present variable vector competence and/or capacity for different pathogens; for instance, *E. canis* is primarily vectored by *Rh. sanguineus* (*s.l.*) tropical lineage [84].

## Conclusions

Data herein reported updates the list of pathogens occurring in ticks and fleas from companion dogs and cats in EA and SEA. By sharing the common environment with humans, these parasitic arthropods could be responsible for the transmission of pathogens to humans (i.e. *R. felis*). Strategies to prevent tick and flea infestations in these animals are fundamental to decrease the risk of transmission of VBPs to animals and humans.

## Data Availability

All data generated or analyzed during this study are included in this published article. Representative newly generated sequences were submitted in the GenBank database under the accession numbers MT499354-MT499356 (*H. canis*), MT499357 (*B. vogeli*), MT499358 and MT499359 (*A. platys*), MT499360 and MT499361 (*E. canis*), MT499362 (*Rickettsia* sp.), MT499363-MT499367 (*R. felis*), MT499368-MT499370 (*R. asembonensis*) and MT499371 (“*Ca.* R. senegalensis”).
